# Successful esophageal endoscopic submucosal dissection with intraoperative release of stenosis due to previous endoscopic submucosal dissection scarring

**DOI:** 10.1002/deo2.87

**Published:** 2022-01-06

**Authors:** Rindo Ishii, Ken Ohata, Rikimaru Sawada, Nao Takeuchi, Marie Kurebayashi, Rin Inamoto, Syunya Takayanagi, Yoshiaki Kimoto, Mako Nohara, Bo Liu, Ryoju Negishi, Yohei Minato, Takashi Muramoto

**Affiliations:** ^1^ Department of Gastrointestinal Endoscopy NTT Medical Center Tokyo Tokyo Japan

**Keywords:** endoscopic submucosal dissection, esophageal squamous cell carcinoma

## Abstract

Endoscopic submucosal dissection (ESD) is the standard endoscopic treatment for early esophageal cancer. Esophageal stricture often occurs at the site of ESD for large lesions. When treating a metachronous lesion appearing at the severe stricture, it may be difficult to negotiate a conventional endoscope through the stricture. Using a thin endoscope may be a useful strategy for such lesions, though ESD using a thin endoscope is challenging because of poor maneuverability. Herein, we report a case of successful ESD for early esophageal cancer at the severe stricture, using a conventional endoscope. A 72‐year‐old man with a previous history of ESD for esophageal cancer and a post‐ESD esophageal stricture was referred to our hospital for metachronous early esophageal cancer. The lesion, 10 mm in diameter, was located at the stricture with a slight distal extension. Conventional endoscopes could not be negotiated through stricture. Therefore, submucosal dissection was performed from the oral to the anal aspect of the lesion, as far as possible. After completion of submucosal dissection of the oral aspect of the lesion and part of the lesion located on the stricture, the severe stricture was released, allowing the passage of conventional endoscope, and ESD of the entire lesion was completed en bloc. Histopathological examination showed squamous cell carcinoma, pT1a‐LPM. Stricture due to scarring may occur during the regeneration process of the defective mucosa, muscularis mucosa, and submucosal layer. Therefore, incision and dissection of the contracted mucosa, mucularis mucosa, and submucosal layer would release the stenosis.

## INTRODUCTION

Endoscopic submucosal dissection (ESD) is the standard endoscopic treatment for early esophageal cancer.^1^ However, esophageal stricture often occurs at the site of ESD for large lesions.^2^ Furthermore, metachronous squamous cell carcinoma (SCC) sometimes develops at the site of strictures after ESD.^3^, ^4^ When treating a metachronous lesion appearing at the severe stricture, it may be difficult to negotiate a conventional endoscope through the stricture. Balloon dilation is commonly performed for post‐ESD stricture in advance in such cases; however, there is a risk of severe tearing of the lesions if the lesion is located on the stricture.^5^ In such cases, the usefulness of a thin endoscope has been reported,^6^ even though ESD using a thin endoscope is challenging because of poor maneuverability.

Herein, we report a case of successful ESD for early esophageal cancer at the site of a severe stricture, only using a conventional endoscope without any dilation.

## CASE REPORT

**FIGURE 1:**
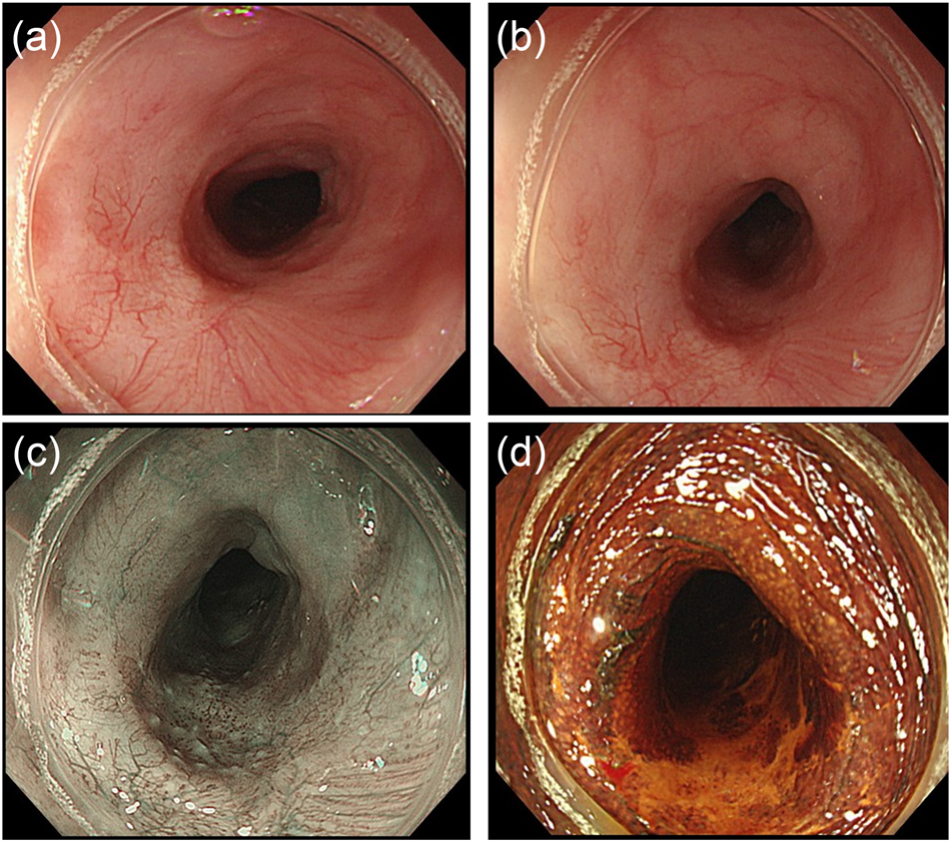
(a) Early esophageal cancer, measuring 10 mm in diameter, in the middle thoracic esophagus. (b) The lesion is located at the site of the post‐endoscopic submucosal dissection (ESD) stricture with a slight distal extension. (c) Narrow‐band imaging of the lesion. (d) Lugol staining of the lesion

**FIGURE 2:**
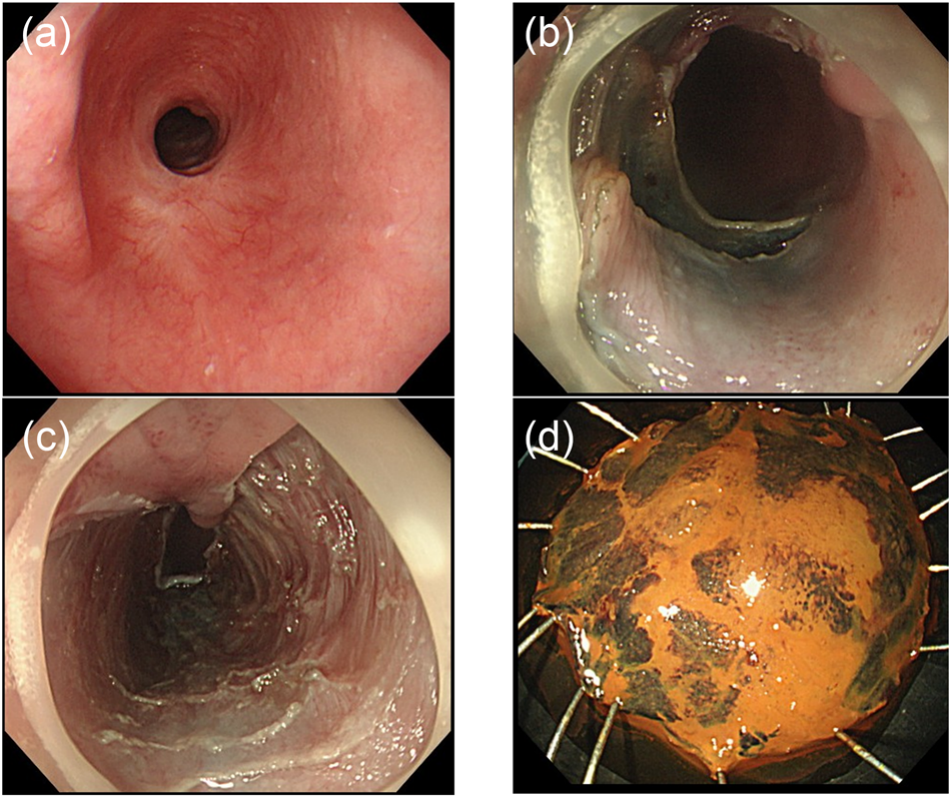
(a) Esophageal stricture caused by a post‐endoscopic submucosal dissection (ESD) scar. (b) The stricture of the esophagus was released when submucosal dissection of the lesion at the oral aspect of the stricture was completed. (c) The ulcer scar after ESD. (d) Resected specimen; en‐bloc resection was achieved

A 72‐year‐old man with a previous history of ESD for esophageal cancer and a post‐ESD esophageal stricture was referred to our hospital for metachronous early esophageal cancer. The patient had received curative resection twice for early‐stage esophageal SCC. The primary tumor was a 30 mm semi‐circumferential lesion and was cured by ESD 8 years ago. The first metachronous tumor at the site of the scar was a 33 mm semi‐circumferential lesion and treated by the same procedure 2 years ago. This time, another metachronous lesion, 10 mm in diameter, was located at the stricture with a slight distal extension. (Figure 1a–d). Conventional endoscopes could not be negotiated through the stricture (Figure 2a). Balloon dilation might hurt the lesion since the lesion was on the stricture. And severe fibrosis was expected under the lesion, thus ESD using a thin endoscope was considered to be extremely difficult. Therefore, we planned to perform ESD with a conventional scope as much as possible, instead of using a thin endoscope. ESD was performed using an Olympus GIF‐H290T endoscope (diameter: 9.9 mm; Olympus, Tokyo, Japan). The whole ESD procedure was performed using a Dual knife (Olympus). The settings of the VIO300D electrical unit (Erbe Elektromedizin, Tübingen, Germany) were EndoCut mode I (effect 2, duration 2, interval 2) for mucosal incision and forced coagulation mode (effect 2, 45 W) for dissection and vessel coagulation. CO_2_ was used as the air supply from the endoscope.

**FIGURE 3:**
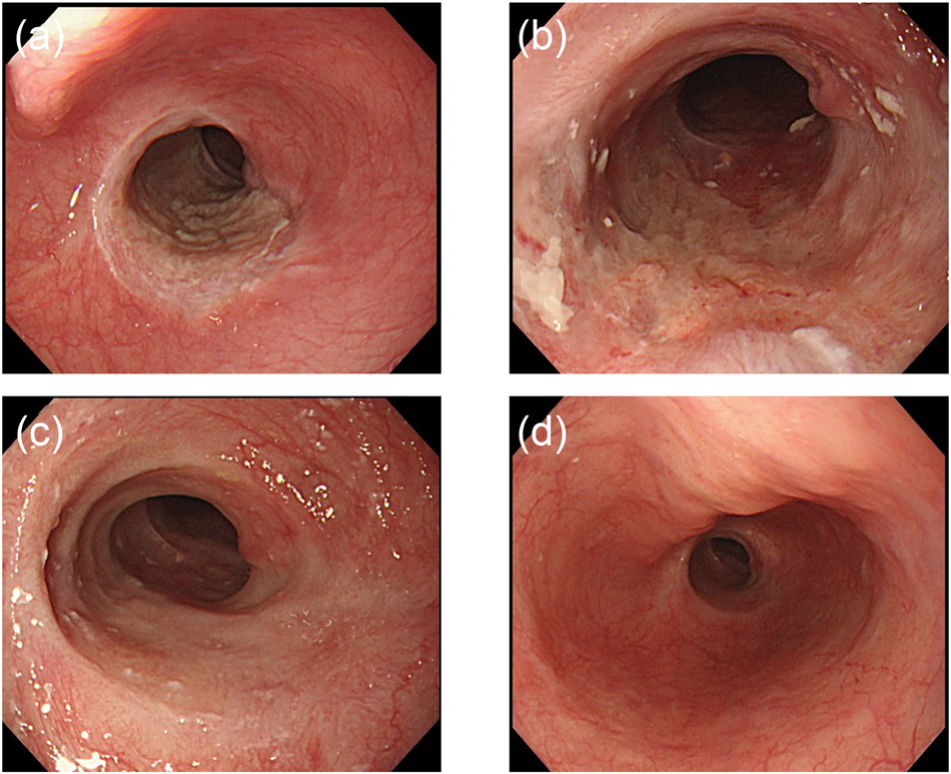
(a) The ulcer floor 1 week post‐ESD. (b) The ulcer floor 1 month post‐ESD. (c) The ulcer floor 2 months post‐ESD. (d) The ulcer floor 6 months post‐ESD

Marking around the lesion beyond the stricture was performed by putting out the device long. Fibrosis of stricture was so severe that little submucosal injection was possible. After a mucosal incision over the oral aspect, submucosal dissection was performed from the oral to the anal aspect of the lesion, as far as possible. Muscularis mucosae were ruptured by submucosal dissection that led to the release of the stricture. The stricture was gradually released as submucosal dissection advanced. After completion of submucosal dissection of the oral aspect of the lesion and part of the lesion located on the stricture, the severe stricture was released, allowing the passage of conventional endoscope (Figure 2b), and ESD of the entire lesion was completed en bloc (Figure 2d), without shifting to a thin endoscope and there was not any complication despite severe fibrosis (Figure 2c). Histopathological examination showed SCC, pT1a‐LPM without lymphovascular infiltration. The entire procedure is shown in the video. All the procedure was performed by one skilled endoscopist who performed more than 1000 cases of ESD. Since there was no remaining submucosal layer of the post ESD ulcer base (Figure 2c), intralesional steroid injection was not performed after ESD to avoid delayed perforation. Oral prednisolone was started with 35mg daily on the third post‐ESD day, tapered gradually (daily 35, 30, 25, 20,15, 10, and 5 mg for 14 days each), and finished after 16 weeks. Follow‐up endoscopy was performed every week and steroid injection was performed four times (1, 2, 3, and 4 weeks after ESD). Six months after ESD, the conventional scope could pass through without difficulty, and the patient remained free of dysphagia (Figure 3a–d).

## DISCUSSION

It has been reported that lesions in >3/4 of the circumferential area were associated with postoperative stricture after esophageal ESD.^2^ The stricture post‐ESD for such lesions sometimes form severe strictures that the conventional scope cannot be negotiated through. The metachronous esophageal cancer sometimes develops at the site of strictures after ESD.^3^, ^4^ If the lesion is on the anal side of the post‐ESD stricture, we can perform balloon dilation or radial incision cutting (RIC) in advance to release the stricture.^7^ Though, in this case, the lesion was at the site of severe stricture, and there is a risk of severe tearing of the lesion if we perform balloon dilation or RIC. We have previously reported successful gastric ESD using a thin endoscope for the lesion with the severe esophageal stricture that a conventional scope cannot be negotiated through. Using a thin endoscope may be a useful method for this case; however, it may be a difficult method due to its poor maneuverability. Using a thin endoscope such as GIF‐H290 (diameter: 8.9 mm) may be another option, though, in this case, GIF‐H290 could not pass through the stricture either. Thus, we considered using a conventional scope as much as possible and shifting to the thin endoscope if we cannot continue to perform the procedure. In this case, we could successfully perform ESD without using a thin endoscope.

In this case, a conventional scope could be negotiated through the stricture after performing a submucosal dissection of the oral aspect and part of the lesion located on the stricture. ESD is a technique that removes the depth limited to the submucosa. It means that no defect is created in the muscular layer.　Stricture due to scarring may occur during the regeneration process of the defective mucosa, muscularis mucosa, and submucosal layer, but not in the muscle layer. Therefore, incision and dissection of the contracted mucosa, mucularis mucosa, and submucosal layer would release the stenosis. In this case, as the incision and dissection of the stenotic area proceeded, the stenosis was gradually released because the tension formed by contracting these layers was released. Nonaka et al. has reported that strictures formed after ESD are considered to comprise spindle‐shaped myofibroblasts arranged in regular concentric layers.^8^ It is also speculated that submucosal dissection disrupts this arrangement, releasing the stricture. If the lesion is longer and narrower than that in this case or located at esophagogastric junction, it may be more difficult, though stricture will release the same process and the procedure may be accomplished in the same way.

Being able to anticipate that the stenosis could be released intraoperatively will help in designing a treatment strategy. Although this strategy may be useful, this therapy is so difficult that only a skilled endoscopist could perform this therapy. There was some report that perform esophageal ESD close to a previous ESD scar,^9^ though this report is attached a video of the whole detailed procedure (Supporting Information).

In conclusion, we report a case of successful ESD for esophageal cancer located at the site of a severe stricture using only a conventional endoscope.

## CONFLICT OF INTEREST

The authors declare that they have no conflict of interest.

## FUNDING INFORMATION

None

## ETHICS STATEMENT

All procedures followed have been performed in accordance with the ethical standards laid down by the Declaration of Helsinki and its later amendments.

## Supporting information

The video shows all the procedures of successful esophageal endoscopic submucosal dissection (ESD) with intraoperative release of stenosis due to previous ESD scarring.
